# Supplementary material for: JUNIPER: Reconstructing Transmission Events from Next-Generation Sequencing Data at Scale
Source: Res Sq. 2025 Mar 27:rs.3.rs-6264999. Preprint. [Version 1] doi: 10.21203/rs.3.rs-6264999/v1 (PMC11975037; doi:10.21203/rs.3.rs-6264999/v1)
Supplement: 1 [file NIHPPRS6264999V1-supplement-1.pdf]

SUPPLEMENTARY FIGURES

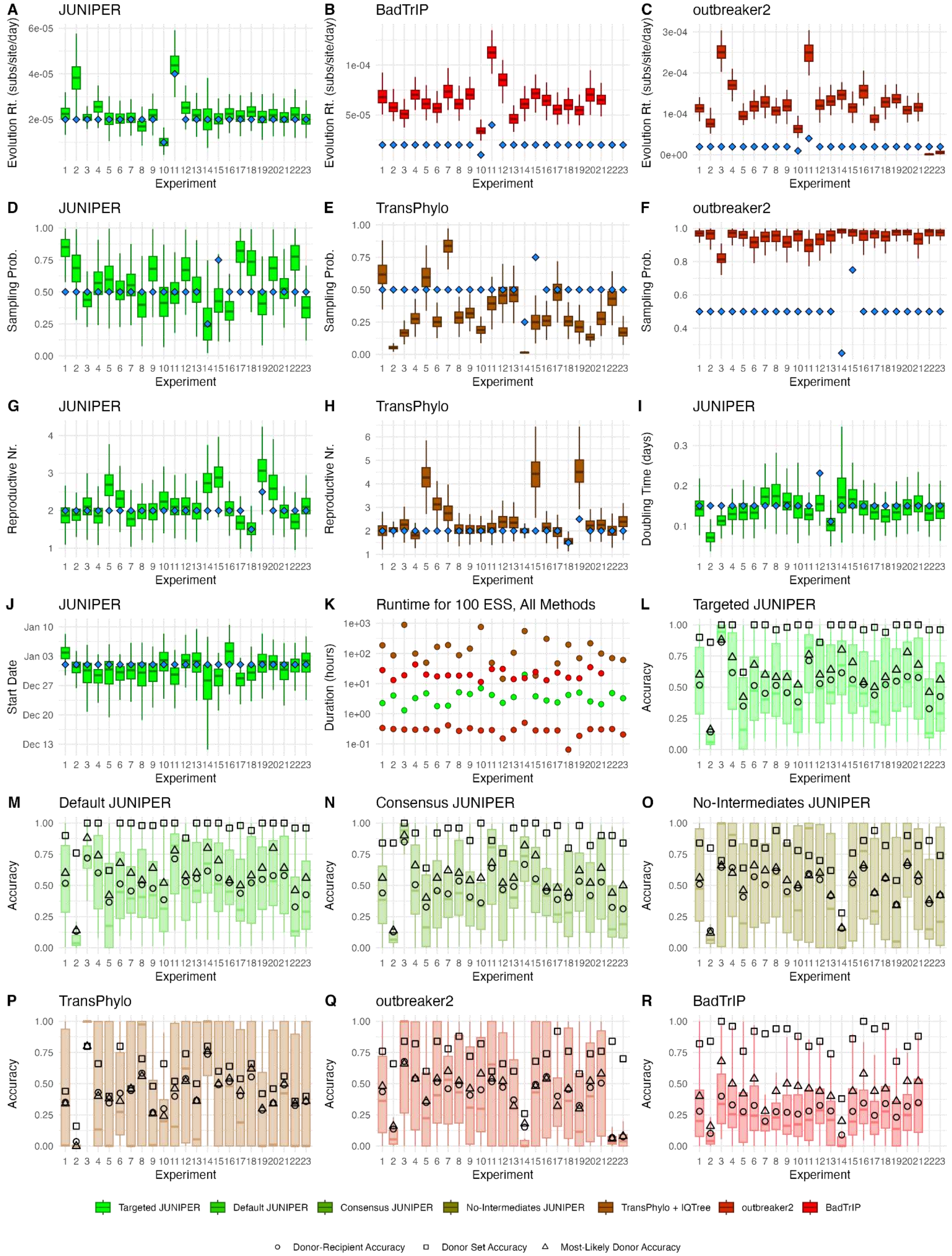

Supplementary Figure S1: Assessing JUNIPER's performance on 23 simulated outbreaks. **(A–H)** Posterior density of the **(A–C)** evolution rate, **(D–F)** sampling rate, **(G–H)** reproductive number, **(I)** within-host effective population size doubling time, and **(J)**, inferred by **(A, D, G, I, J)** JUNIPER, **(B)** BadTrIP, **(C, F)** outbreaker2, and **(E, H)** TransPhylo. The true parameter value is shown in blue. **(K)** Runtime to reach an effective sample size of 100 for JUNIPER, TransPhylo, outbreaker2, and BadTrIP. **(L–R)** Distribution of posterior probabilities assigned to ground-truth direct or indirect transmission links (boxplots), donor-recipient accuracy (circles), donor set accuracy (squares), and most likely donor accuracy (triangles) for **(L)** JUNIPER with correctly-specified input parameters, **(M)** JUNIPER with default input parameters, **(N)** JUNIPER without inferring unsampled intermediates, **(O)** JUNIPER without access to within-host variation data, **(P)** IQ-TREE and TransPhylo, **(Q)** outbreaker2, and **(R)** BadTrIP.

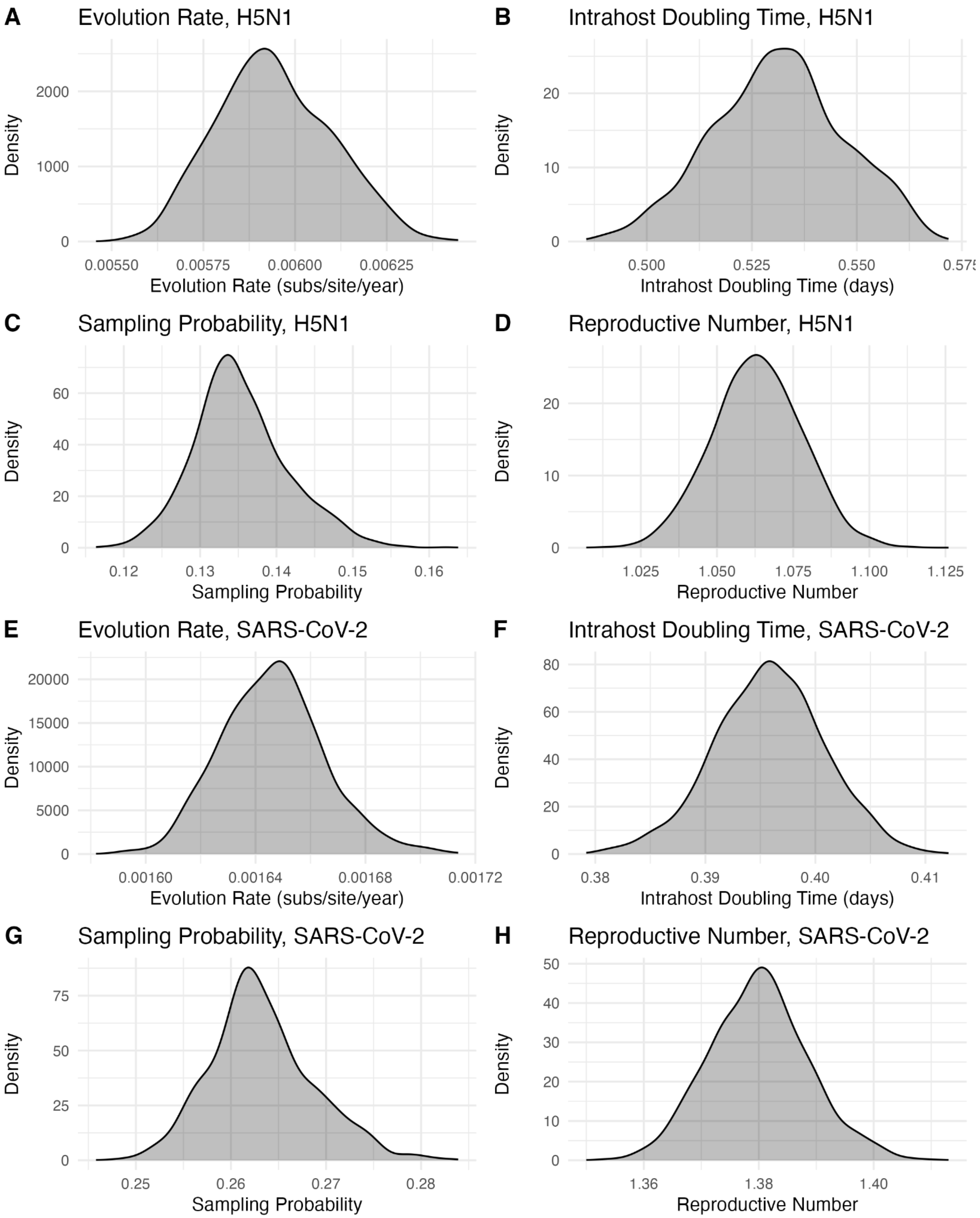

Supplementary Figure S2: Posterior parameter densities for the bovine H5N1 and Massachusetts SARS-CoV-2 datasets. Posterior densities of the **(A, E)** evolution rate, **(B, F)**

doubling time for the within-host effective population size, **(C, G)** sampling probability, and **(D, H)** reproductive number for **(A–D)** H5N1 and **(E–H)** SARS-CoV-2.

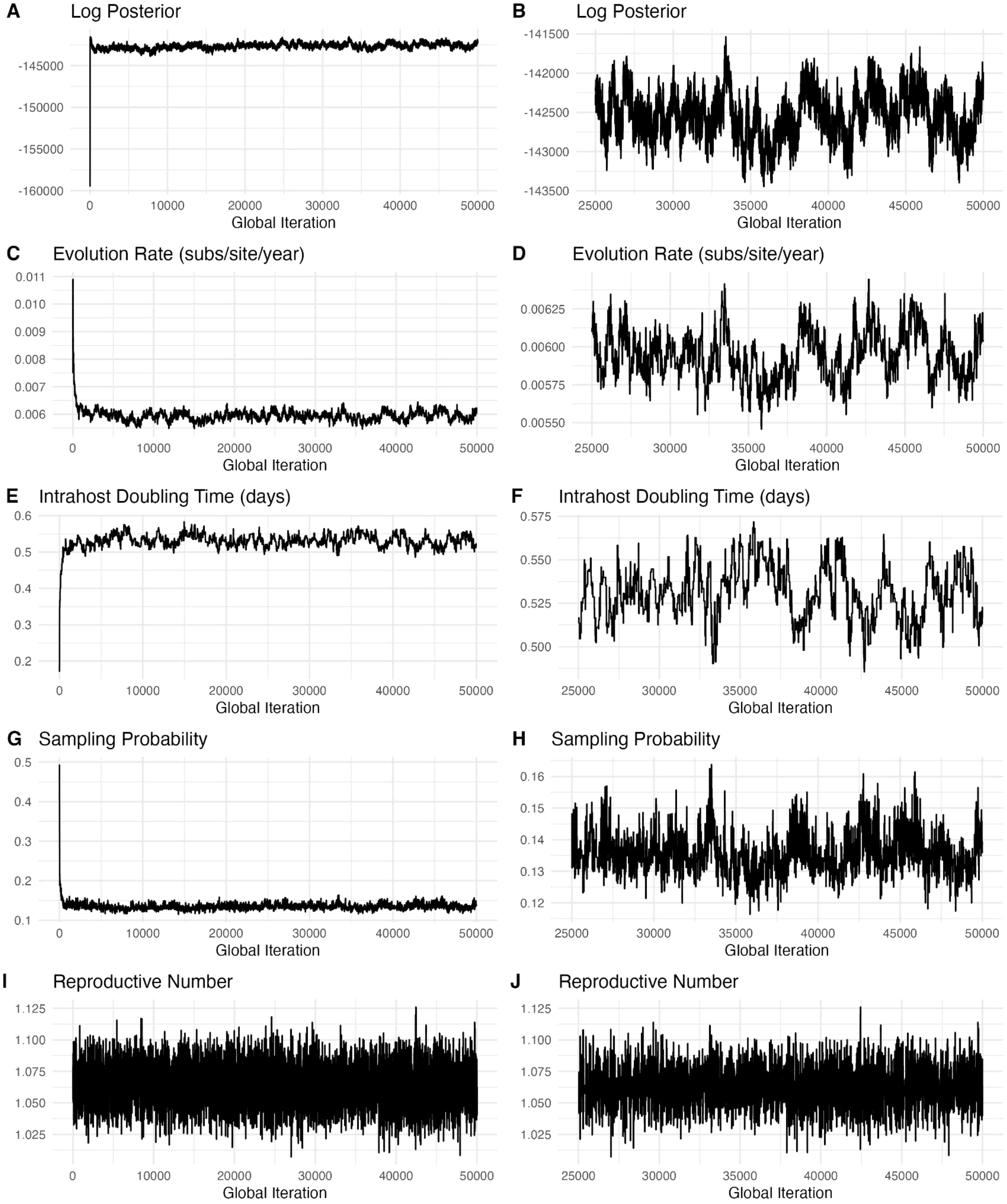

Supplementary Figure S3: MCMC diagnostics for 1,519 H5N1 cases in cattle. Values of the **(A–B)** log posterior, **(C–D)** evolution rate, **(E–F)** within-host effective population size doubling time, **(G–H)** sampling rate, and **(I–J)** reproductive number for each MCMC iteration. Panels on the right-hand side of the figure show iterations after a 50% burnin period; panels on the left-hand side show all iterations. The effective sample sizes based on each trace post-burnin were 68, 53, 60, 289, and 2,480, respectively.

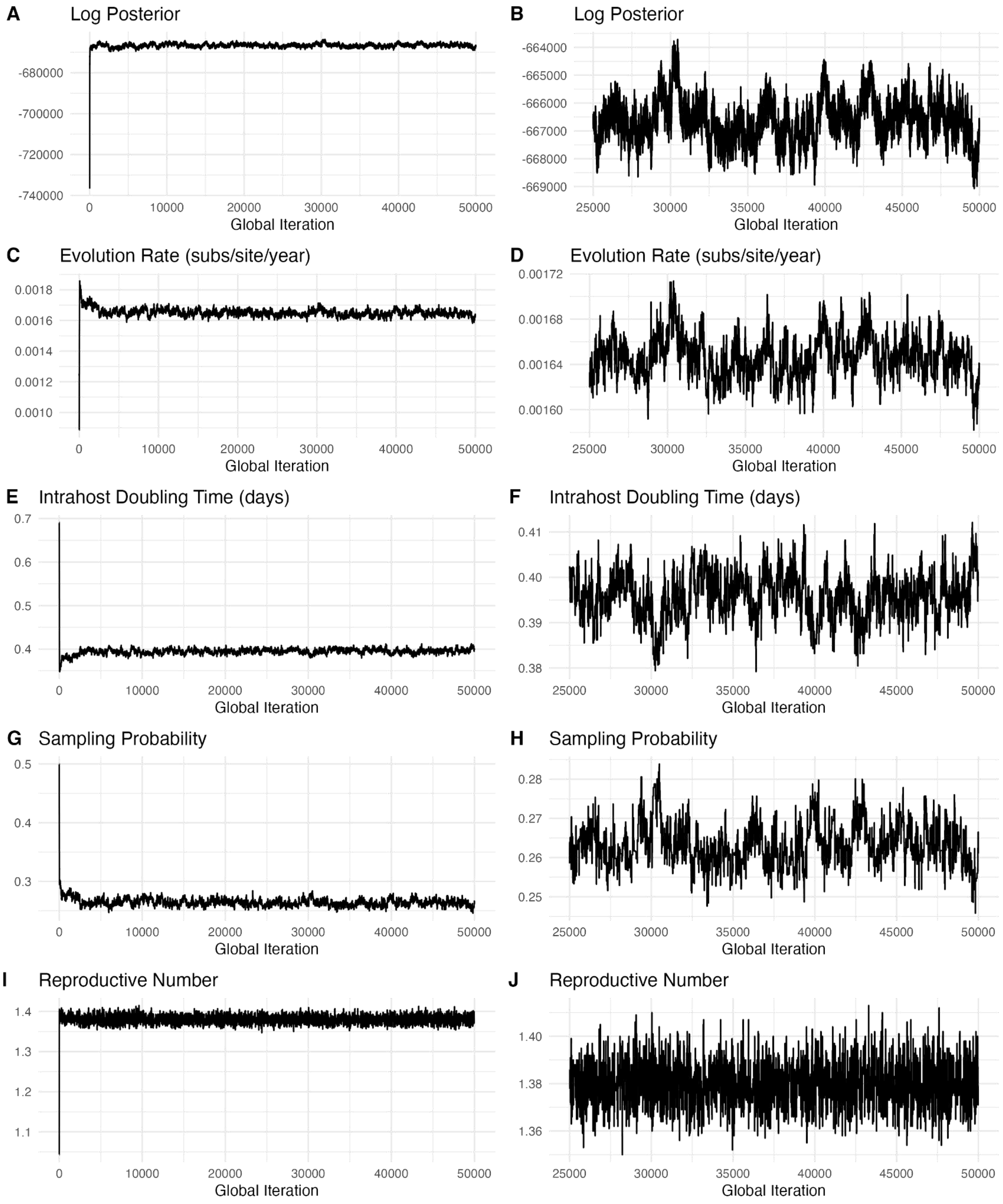

Supplementary Figure S4: MCMC diagnostics for 13,570 Massachusetts SARS-CoV-2 genomes. Values of the **(A–B)** log posterior, **(C–D)** evolution rate, **(E–F)** within-host effective population size doubling time, **(G–H)** sampling rate, and **(I–J)** reproductive number for each MCMC iteration. Panels on the right-hand side of the figure show iterations after a 50% burnin

period; panels on the left-hand side show all iterations. The effective sample sizes based on each trace post-burnin were 82, 117, 139, 173, and 1,534, respectively.

SUPPLEMENTARY TABLES

Experiment #	μ_g	σ_g^2	μ_s	σ_s^2	μ	β	π	ρ_s	R	ψ	γ
1	5	5	5	5	2.00E-05	ln(100)	0.5		2	0.5	1
2	2.5	5	5	5	2.00E-05	ln(100)	0.5		2	0.5	1
3	10	5	5	5	2.00E-05	ln(100)	0.5		2	0.5	1
4	5	2.5	5	5	2.00E-05	ln(100)	0.5		2	0.5	1
5	5	10	5	5	2.00E-05	ln(100)	0.5		2	0.5	1
6	5	5	2.5	5	2.00E-05	ln(100)	0.5		2	0.5	1
7	5	5	10	5	2.00E-05	ln(100)	0.5		2	0.5	1
8	5	5	5	2.5	2.00E-05	ln(100)	0.5		2	0.5	1
9	5	5	5	10	2.00E-05	ln(100)	0.5		2	0.5	1
10	5	5	5	5	1.00E-05	ln(100)	0.5		2	0.5	1
11	5	5	5	5	4.00E-05	ln(100)	0.5		2	0.5	1
12	5	5	5	5	2.00E-05	ln(20)	0.5		2	0.5	1
13	5	5	5	5	2.00E-05	ln(500)	0.5		2	0.5	1
14	5	5	5	5	2.00E-05	ln(100)	0.25		2	0.5	1
15	5	5	5	5	2.00E-05	ln(100)	0.75		2	0.5	1
16	5	5	5	5	2.00E-05	ln(100)	0.5	0.25	2	0.5	1
17	5	5	5	5	2.00E-05	ln(100)	0.5	0.75	2	0.5	1
18	5	5	5	5	2.00E-05	ln(100)	0.5		1.5	0.5	1
19	5	5	5	5	2.00E-05	ln(100)	0.5		2.5	0.5	1
20	5	5	5	5	2.00E-05	ln(100)	0.5		2	0.25	1
21	5	5	5	5	2.00E-05	ln(100)	0.5		2	0.75	1
22	5	5	5	5	2.00E-05	ln(100)	0.5		2	0.5	0.8
23	5	5	5	5	2.00E-05	ln(100)	0.5		2	0.5	0.9

Supplementary Table S1: Parameters of each experiment, where μ_g and σ_g^2 are the mean and variance of the Gamma-distributed generation interval, respectively; μ_s and σ_s^2 are the mean and variance of the Gamma-distributed sojourn interval, respectively; μ is the evolution rate in substitutions per site per day; β is the growth rate per day of the within-host viral population (i.e.

the within-host effective population size t days after inoculation is $\exp(\beta t)$); π is the probability that a case is sampled and sequenced; ρ_s (if specified) is the probability that a case is sampled and sequenced given that its its infector is sampled and sequenced; the offspring distribution is Negative Binomial with mean R and variance R/ψ; and γ is the fraction of non-ambiguous sites in sequencing data. Values differing from those used in experiment 1 (i.e. the default) are highlighted in orange. All genomes had a length of 10,000 nucleotides.

From	*To*	*Supporting Evidence from [53]*
P1	P3	"On review of the timeline, it was noticed that the first outpatient case (P1) and the person who would become the first inpatient case (P3) were both in the Emergency Department on 9 March. A more detailed investigation of events of that day uncovered that they were in the ED at overlapping times, were in close proximity to one another, and were attended to by the same medical officer."
P3	HW1	"On review of [HW1's] shift patterns and patient allocation, it was noted that she was directly responsible for the care of P3 in CICU on the night shift of 12 Mar–13 March. Based on the understanding that people can be infectious 1-3 days prior to symptom onset, this link provides strong circumstantial evidence that P3 was infectious at this time and transmitted to the CICU nurse."
P3	P4, P7, P14	"On the assumption that P3 was infectious throughout her time on MW1 (13-16 Mar), there were five other cases who may have been exposed during that time period. This includes a 46-year-old female (P4) who was in the bed directly opposite (in room 12), and four male patients (P6, P7, P14, X4) who were co-located in a room down the corridor (room 15)." [Note: P6 and X4 were not sequenced.]
P4	HW4	"A professional nurse on this ward (HW4) became symptomatic with fever and flu-like symptoms at the end of her shift on 23 March and subsequently tested positive on 29 March. This nurse worked directly with P4 on 23 March, when P4 was coughing, and performed tasks including nebulisation." [Note: all methods from our analysis, as well as the findings of San et al. [43], refute this link.]
HW4	P11, P15	"[HW4] was signed off work until 27 March and when she returned to work on 27 March she was still symptomatic with cough, fever and sore throat, but she continued to work day shifts on 27 & 28 March. On those days she worked directly with P11 and P15."
P3	P10	"On 16 April, patient P3 was discharged from St. Augustine's Hospital to the Bill Buchanan Association for the Aged in Morningside, a nursing home with 210 residents. She was there until her readmission to St.

		Augustine's on 22 March. By 31 March, we understand that four other residents at the home were diagnosed with COVID-19, including three women who had shared the sick bay with P3 and one woman who stayed in a separate unit and only visited P3 (as they were friends from their residential home). One of the cases from the sick bay was admitted to St. Augustine's on 31 March (patient P10)."

Supplementary Table S2: Epidemiological evidence supporting the putative transmission links identified in the South Africa SARS-CoV-2 nosocomial study.
